# Dynamic peripheral immune monitoring during checkpoint blockade: a framework for interpreting response, toxicity and resistance

**DOI:** 10.3389/fimmu.2026.1902709

**Published:** 2026-08-03

**Authors:** Lina Gao, Yinghua Jin

**Affiliations:** Department of Oncology, the Fourth Affiliated Hospital of Soochow University, Medical Center of Soochow University, Suzhou Dushu Lake Hospital, Suzhou, Jiangsu, China

**Keywords:** cytokine, dynamic immune monitoring, immune checkpoint inhibitors, immune-related adverse events, immunotherapy resistance, peripheral immune biomarkers

## Abstract

Immune checkpoint inhibitors (ICIs) have reshaped cancer treatment, but their clinical benefit remains variable. Many patients show primary or acquired resistance, and immune-related adverse events (irAEs) can limit treatment or complicate clinical decisions. Tissue-based biomarkers have improved patient selection in selected settings. These include programmed death-ligand 1 (PD-L1) expression, tumor mutational burden, microsatellite instability/mismatch repair deficiency, tumor-infiltrating lymphocytes and T-cell-inflamed gene signatures. Yet these markers are mostly static and tumor-centered. They do not fully capture host immune competence, systemic inflammation, treatment-induced immune changes or toxicity-prone immune activation. Peripheral immune biomarkers may help address this gap. Blood-based markers are repeatable and can be assessed across treatment, making them useful for longitudinal monitoring during checkpoint blockade. In this review, we discuss peripheral biomarkers as dynamic readouts rather than isolated predictors of response, toxicity or resistance. For narrative synthesis, we group these signals into four heuristic interpretive patterns developed for this review rather than established biological states or clinically validated patient categories: immune-cold resistance, effective anti-tumor activation, toxicity-prone inflammatory amplification and inflamed but ineffective resistance. We review several layers of peripheral monitoring, including immune-cell dynamics, T-cell receptor repertoire changes, cytokine and chemokine networks, humoral signals, eosinophilic inflammation and circulating tumor burden markers. We discuss how these readouts can be interpreted across key clinical windows. These include baseline, early on-treatment assessment, first imaging, irAE onset, progression and rechallenge. Peripheral monitoring should complement tissue biomarkers, imaging and clinical judgment. Its clinical translation will require standardized sampling, assay harmonization, integrated interpretation and prospective validation.

## 1 Introduction

Immune checkpoint inhibitors (ICIs) have reshaped cancer therapy by enabling durable immune-mediated tumor control across multiple malignancies. By blocking programmed cell death protein 1 (PD-1), programmed death-ligand 1 (PD-L1) or cytotoxic T-lymphocyte-associated protein 4 (CTLA-4), these agents restore or amplify endogenous anti-tumor immunity. Their effects therefore depend not only on tumor-intrinsic features but also on the host immune system’s capacity to recognize, mobilize and regulate an effective response (1–3).

Clinical benefit, however, remains variable. Many patients have primary resistance, and some who initially respond later develop acquired resistance (4). Immune-related adverse events (irAEs) further complicate treatment and require organ- and severity-specific management (5, 6). Although therapeutic response and irAEs may share upstream immune activation, they are distinct outcomes and should not be interpreted interchangeably (7). Together, these challenges create a need for biomarkers that track how anti-tumor and inflammatory immune responses evolve during treatment.

Current tissue-based biomarkers provide important but incomplete information. PD-L1 expression, T-cell-inflamed gene expression signatures, tumor mutational burden (TMB), and microsatellite instability or mismatch repair deficiency (MSI/dMMR) help characterize tumor immunogenicity and guide treatment in selected settings (8–10). However, a tissue sample offers a spatially and temporally limited view of a dynamic, systemic response. It cannot fully capture host immune competence, treatment-induced immune changes or immune activation associated with toxicity.

Serial blood sampling offers a practical complement to tissue assessment because it can track immune changes over time with relatively low procedural risk. Studies of early T-cell reinvigoration relative to tumor burden, baseline and on-treatment cytokine changes, T-cell receptor repertoire dynamics and circulating immune-cell composition illustrate the potential clinical value of this approach (11–14). Yet no peripheral signal is specific in isolation: its meaning depends on timing, the direction and magnitude of change, and clinical context, including treatment response, tumor progression, infection and tissue injury.

**Figure 1:**
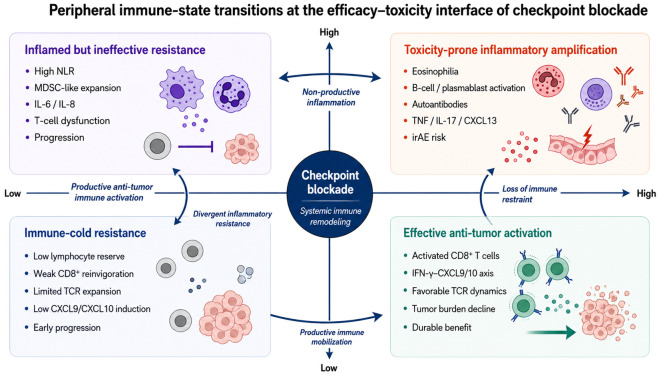
Peripheral immune-state transitions at the efficacy-toxicity interface of checkpoint blockade. This hypothesis-generating framework summarizes four heuristic interpretive patterns developed for this review: immune-cold resistance, effective anti-tumor activation, toxicity-prone inflammatory amplification and inflamed but ineffective resistance. The axes represent productive anti-tumor immune activation and inflammatory or toxicity propensity. The framework is intended to guide longitudinal interpretation of peripheral immune signals rather than define clinically validated patient categories.

In this review, we examine peripheral immune biomarkers as longitudinal readouts of systemic immune activity during checkpoint blockade. We consider how cellular, clonal, soluble, humoral and tumor-burden signals may inform the interpretation of response, toxicity and resistance across clinically relevant treatment windows. We also use four overlapping interpretive patterns as a heuristic narrative framework, not as established biological states or clinically validated patient categories. **Figure 1** summarizes this framework and its intended use.

## 2 Static tissue biomarkers define tumor immunogenicity

Tissue-based biomarkers established that tumor immunogenicity and local immune contexture influence sensitivity to checkpoint blockade. PD-L1 expression, TMB, MSI/dMMR, tumor-infiltrating lymphocytes (TILs) and IFN-γ-related T-cell-inflamed gene expression signatures have informed patient selection and the biology of ICI response (8–10, 15). In appropriate clinical settings, these markers remain central to treatment decisions. Their main limitation is not lack of value, but the restricted spatial and temporal view provided by a tissue sample. **Supplementary Table 1** summarizes their clinical roles and their limitations for systemic monitoring.

PD-L1 is the most widely used tissue biomarker in clinical immuno-oncology. Expression on tumor or immune cells can mark an inflamed or adaptively resistant local context and has treatment-specific predictive value in selected tumor types and regimens (1, 8). Interpretation is complicated by interlesional heterogeneity, temporal variation, assay differences, scoring systems and uncertain cutoffs. A single result cannot resolve immune heterogeneity across metastatic sites or show whether peripheral effector immunity will be mobilized during treatment.

TMB and MSI/dMMR assess tumor antigenic potential rather than local immune contexture. High TMB can increase the probability of neoantigen generation, and MSI-H/dMMR tumors are often immunogenic and sensitive to PD-1 blockade (9, 10, 17, 18). Antigenic potential does not, however, ensure productive anti-tumor immunity. Tumor PD-L1 expression and TMB provide partly independent information, and TMB thresholds associated with outcome vary across cancer types (16, 18). These genomic measures also provide little direct information about host immune reserve or susceptibility to immune-mediated injury.

TILs, tertiary lymphoid structures and IFN-γ/T-cell-inflamed signatures capture a more functional dimension of tumor immunity. They reflect immune infiltration, cytotoxic activity, chemokine production and adaptive immune resistance within the tumor microenvironment (15, 19–21). Such features may identify tumors that are already immunologically visible and therefore more permissive to PD-1/PD-L1 blockade. Even so, they offer limited insight into systemic immune changes during treatment.

Tissue biomarkers therefore answer a different question from serial peripheral monitoring: they characterize whether a sampled lesion is compatible with immune recognition, but do not show how the host response changes over time. This distinction is particularly relevant to irAEs, which arise from systemic or organ-specific immune dysregulation rather than tumor-localized activity alone (6, 22, 23). Serial blood-based readouts can complement, but not replace, tissue assessment.

## 3 Peripheral immune biomarkers as systemic immune-state readouts

Peripheral blood permits repeated assessment of treatment-induced immune changes with relatively low procedural risk. Because response, resistance and irAEs evolve during therapy, peripheral measurements are better treated as time-dependent readouts than as fixed baseline predictors. Response-associated T-cell changes (11, 14, 25), toxicity-associated T-cell features (24), cytokine associations (12) and circulating myeloid-derived suppressor cells (MDSCs) linked to poorer outcomes (26) illustrate why timing and clinical context matter. An inflammatory cytokine, for example, may accompany effector activation, myeloid-driven resistance or tissue injury, and its interpretation depends on the accompanying cellular profile, tumor burden and evidence of organ injury.

Blood-based assays differ in accessibility and biological resolution. Routine cell counts and inflammatory indices are widely available but nonspecific and have been studied as irAE-risk markers (27, 28). Immune-cell profiling, cytokine and chemokine panels, autoantibody testing, T-cell receptor (TCR) sequencing and plasma multi-omics provide more detail, but assay-specific validation remains necessary. Cytokine syntheses show heterogeneous sampling and analyte selection (12), while MDSC studies require consistent marker definitions and cutoffs (26).

**Figure 2:**
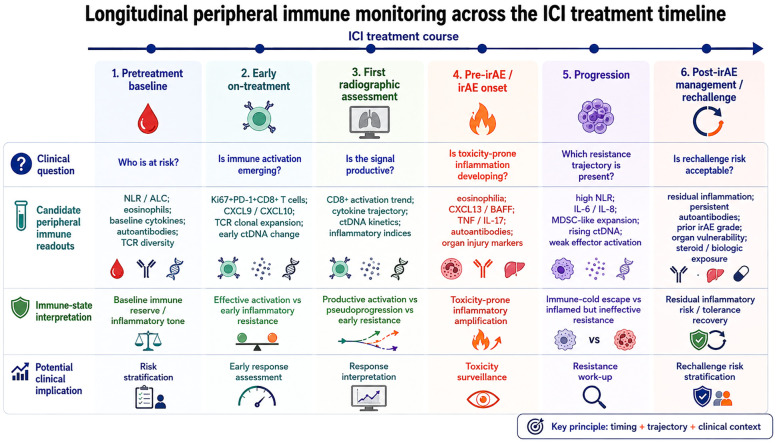
Longitudinal peripheral immune monitoring across the ICI treatment timeline. This figure maps peripheral immune monitoring onto clinically meaningful treatment windows, including pretreatment baseline, early on-treatment immune activation, first radiographic assessment, pre-irAE or irAE onset, progression and rechallenge. Candidate readouts should be interpreted in relation to sampling time, the direction and magnitude of change, and clinical context rather than as isolated baseline predictors.

Candidate sampling windows include baseline, early treatment and first imaging (11, 14, 25), irAE onset (24, 31), progression and rechallenge (61–63). **Figure 2** summarizes these longitudinal monitoring windows across the ICI treatment timeline. Measurements at these points should be interpreted with clinical status, tumor burden and organ-injury markers rather than in isolation. **Table 1** summarizes representative patterns; these patterns supplement tissue biomarkers, imaging and clinical judgment rather than replace them.

**Table 1:** Four heuristic interpretive patterns during checkpoint blockade.

Pattern	Cellular and clonal features	Soluble inflammatory features	Tumor-burden dynamics	Interpretation and clinical relevance
Immune-cold resistance	Delayed or absent early proliferating PD-1+ CD8+ responses (25); low reinvigoration relative to tumor burden (11); failure to develop favorable on-treatment clonality (13).	No established peripheral cytokine pattern; weak inflammatory remodeling alone warrants cautious interpretation.	Persistent or rising ctDNA (68–70).	Limited immune engagement or primary resistance when concordant with imaging and tissue findings; may prompt resistance evaluation or alternative strategies.
Effective anti-tumor activation	Early activated or proliferating PD-1+ CD8+ T-cell expansion (11, 25); favorable TCR clonality (13), tumor-linked clones (29), or neoantigen-specific responses (30).	Moderate inflammatory remodeling may accompany response, but no single circulating cytokine establishes productive anti-tumor activation.	Declining ctDNA (68–70).	Productive activation with objective or durable disease control; may contextualize early imaging and support continued treatment with ongoing toxicity surveillance.
Toxicity-prone inflammatory amplification	Activated CD4 memory or TCR features in melanoma (24); IL-17A+ CD4 expansion in melanoma (31); B-cell or plasmablast changes (35); irAE-associated eosinophilia in NSCLC (32, 38); broad CD8 clonal expansion before ipilimumab toxicity (39).	IL-17/type III activation at irAE onset in melanoma (31); serum IFN-γ associated with irAEs in NSCLC (32).	Variable tumor-burden change; irAE occurrence is not a validated surrogate of tumor control (7, 45–48).	Increased irAE risk without implied efficacy; may warrant organ-specific evaluation (5, 22, 55) and individualized interruption or rechallenge planning (61–63).
Inflamed but ineffective resistance	Circulating MDSCs associated with poorer ICI outcomes (26); nonspecific neutrophil-skewed indices; absent or non-tumor-linked clonal expansion requiring tumor-reactivity validation.	Rising or high IL-8 associated with progression or poor outcome (33, 42); high IL-6 associated with poor outcomes in gastric or esophageal cancer (40).	Persistent or rising ctDNA (68–70).	Inflammatory resistance when systemic inflammation lacks tumor control; may prompt resistance evaluation or trial stratification, but not escalation based on inflammation alone.

This table presents heuristic interpretive patterns for longitudinal peripheral monitoring during ICI therapy. The patterns are overlapping narrative constructs, not validated patient categories or stand-alone treatment rules. Interpretation requires temporal concordance across immune measures, tumor-burden dynamics, imaging, organ-injury findings and clinical context. Tumor-site TGF-β-associated immune exclusion is not included as a peripheral feature and is cited only as complementary tissue evidence (43, 44; ref. 44 preclinical). Abbreviations: CD, cluster of differentiation; ctDNA, circulating tumor DNA; ICI, immune checkpoint inhibitor; IFN, interferon; IL, interleukin; irAE, immune-related adverse event; MDSC, myeloid-derived suppressor cell; NSCLC, non-small cell lung cancer; PD-1, programmed cell death protein 1; TCR, T-cell receptor; TGF-β, transforming growth factor-β.

## 4 Immune-cell and clonal dynamics during checkpoint blockade

Peripheral immune-cell profiling describes which circulating populations expand, contract or change phenotype during checkpoint blockade. In melanoma treated with anti-PD-1 therapy, high-dimensional profiling identified a monocyte-associated signature linked to clinical response, illustrating that host immune states can be detected in blood (14). Interpretation should remain tied to sampling time and the measured phenotype; tumor burden is particularly important for response-associated T-cell readouts (11), while tumor-blood clonal overlap improves confidence in tumor-directed activity (29).

Effector T-cell mobilization is an early sign of immune engagement, particularly when activated or proliferating CD8+ subsets expand after treatment. Huang et al. found that reinvigoration of circulating PD-1+ CD8+ T cells relative to pretreatment tumor burden was associated with response to anti-PD-1 therapy (11). The ratio is informative because T-cell expansion alone does not establish tumor-directed activity. Expansion without tumor regression may reflect bystander activation, poor tumor trafficking or terminal dysfunction. Evidence of declining tumor burden (11), functional activity or tumor-blood and neoantigen linkage (29, 30) is therefore needed before a peripheral T-cell response is interpreted as tumor specific.

CD4+ T-cell and regulatory programs provide additional context rather than stand-alone prediction. Helper subsets may support CD8+ effector function, B-cell activation and cytokine remodeling, whereas Th17-skewed programs may contribute to inflammatory toxicity (31), and early B-cell or plasmablast changes may accompany humoral autoimmunity (35). Conversely, increased regulatory T-cell activity may suppress anti-tumor immunity, while loss of regulatory control may facilitate immune-mediated tissue injury. The same regulatory axis can therefore influence efficacy or toxicity depending on timing, tissue context and the balance between effector and suppressive programs.

Myeloid-skewed inflammation represents a different cellular pattern. Higher circulating MDSC levels are associated with poorer ICI outcomes across solid tumors (26), whereas serum IL-8 decreases with response and increases with progression in melanoma and NSCLC (33). Routine neutrophil and neutrophil-to-lymphocyte ratio (NLR) changes are clinically accessible but nonspecific; infection, corticosteroids, comorbidity and tumor burden should be considered as alternative explanations.

Humoral, eosinophilic and selected T-cell changes may instead indicate susceptibility to immune toxicity. B-cell activation, plasmablast expansion and autoantibody changes can accompany loss of tolerance (34–36), while eosinophilia or serum IFN-γ has been associated with irAEs in NSCLC (32, 38). In melanoma, longitudinal T-cell characteristics were associated with severe irAEs during checkpoint blockade (24). Tumor-local B-cell and tertiary lymphoid features may instead accompany response (19), underscoring why none of these signals is uniformly favorable or unfavorable. Interpretation requires the concurrent tumor response, inflammatory profile and organ-specific evidence of injury.

TCR repertoire analysis adds a clonal dimension to peripheral immune monitoring. Diversity and clonality features have been associated with benefit in NSCLC and renal cell carcinoma, with tumor-sharing clones identified in responding renal cell carcinoma (13, 29). Expansion of neoantigen-specific circulating CD8+ populations supports tumor-directed activity in urothelial carcinoma (30), whereas broad CD8+ clonal expansion can precede ipilimumab toxicity (39). Peripheral clonal expansion therefore does not automatically indicate tumor reactivity; expanded clones may be tumor-specific, virus-specific, bystander, tissue-reactive or autoreactive. At present, TCR repertoire analysis is best viewed as a mechanistic and translational tool rather than a clinically actionable assay, particularly when tumor-blood pairing and antigen-specific validation are unavailable.

Cellular and clonal measurements are complementary rather than mutually exclusive. Effector mobilization, suppressive myeloid remodeling and humoral or eosinophilic activation may coexist or change within the same patient. Their main contribution is to distinguish forms of immune engagement; they do not by themselves prove tumor response, resistance or impending toxicity.

## 5 Cytokine and chemokine networks linking response, resistance and toxicity

**Figure 3:**
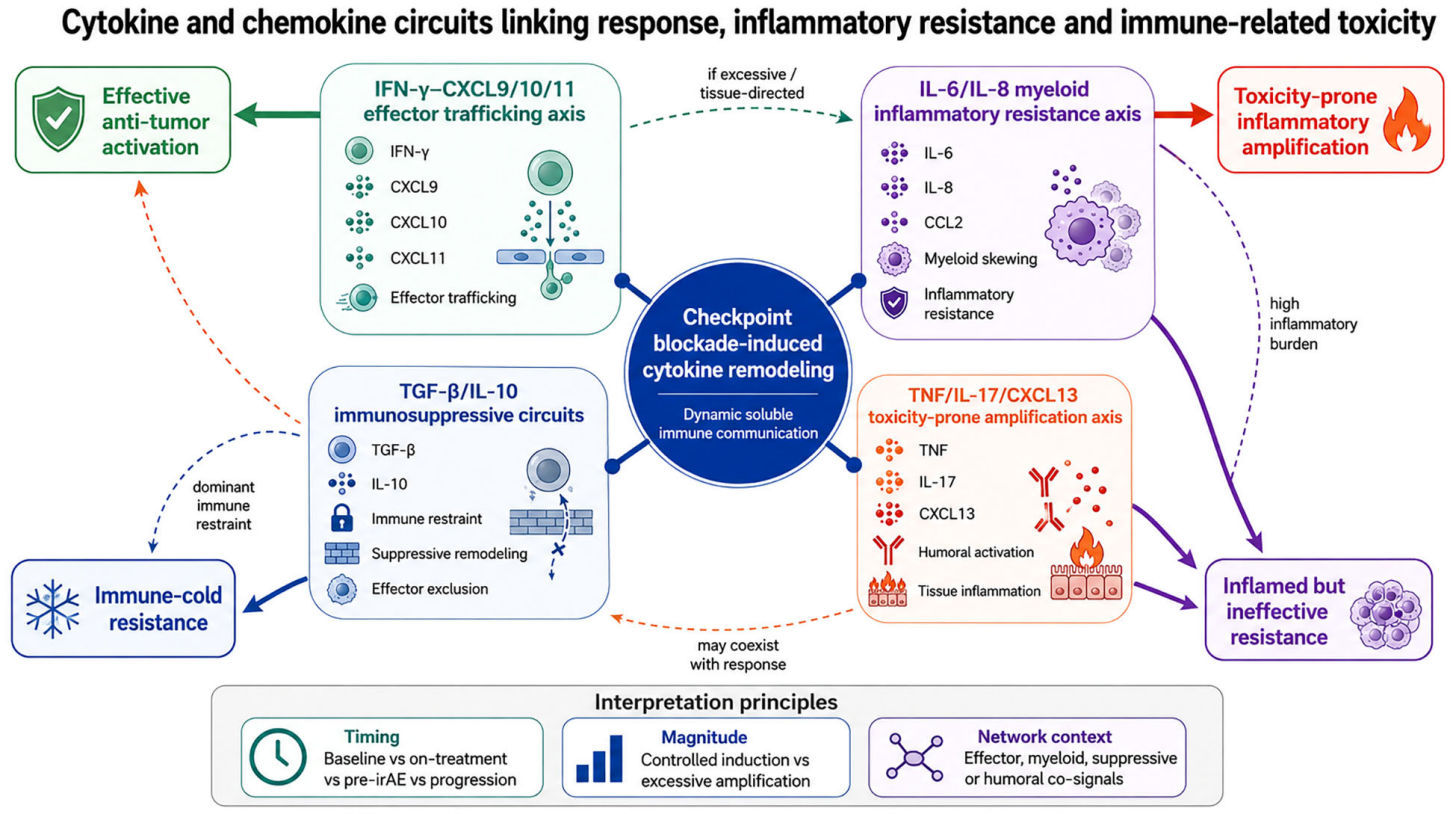
Cytokine and chemokine circuits linking response, inflammatory resistance and immune-related toxicity. This conceptual figure illustrates cytokine and chemokine networks as functional modules rather than isolated analytes. The IFN-γ-CXCL9/10/11 axis, IL-6/IL-8-associated myeloid inflammation, tumor-site TGF-β immune exclusion, and IL-17/type III, B-cell/autoantibody and eosinophil-associated toxicity signals should be interpreted according to timing, magnitude, tumor type, organ context and the supporting evidence described in the text.

Cytokines and chemokines add a soluble, functional layer to peripheral monitoring. Across studies, single baseline cytokines have not shown consistent specificity: IL-6 and IL-8 are associated with ICI outcomes, while the direction of the IL-6 association with irAEs differs across cohorts (12, 40, 41). Interpretation is more informative at the network level: which signals change, when they change and whether they coincide with effector-cell mobilization, tumor burden, suppressive myeloid activity or organ injury. **Figure 3** summarizes the principal networks discussed below.

The IFN-γ–CXCL9/10/11 axis links T-cell activation to CXCR3-dependent effector trafficking. In tumor tissue, this network supports antigen presentation and cytotoxic activity but can also induce PD-L1-mediated adaptive resistance (8, 15). In blood, early proliferating or reinvigorated PD-1+ CD8+ responses are most informative when calibrated to tumor burden and clinical outcome (11, 25); these studies do not establish circulating IFN-inducible chemokines as stand-alone response markers.

Peripheral IFN-γ-related signals are therefore not uniformly favorable. A tumor T-cell-inflamed gene-expression profile may be necessary but not sufficient for benefit (15), while higher serum IFN-γ was associated with irAEs and outcomes in one advanced NSCLC cohort (32). These findings are associations rather than evidence that the same IFN-related program causes tumor control or tissue injury, and peripheral changes should be interpreted with tumor response and organ-specific clinical findings.

Circulating MDSCs and IL-8/CXCL8-associated inflammation can accompany poor ICI outcomes. Lower circulating MDSC levels are associated with better progression-free and overall survival across solid tumors (26), while serum IL-8 decreases with response and increases with progression in melanoma and NSCLC (33), and high IL-8 is associated with poorer response and survival across studies (12, 42). IL-6 findings vary by tumor type and clinical context, including opposing associations with irAEs and poor outcomes in different cohorts (40, 41). Infection, corticosteroids, comorbidity, tissue injury and tumor burden can confound these measurements. When myeloid inflammation coincides with weak T-cell reinvigoration and no tumor-burden decline, it more strongly supports inflammatory resistance than productive immunity.

Transforming growth factor-β (TGF-β) represents an immunosuppressive cytokine circuit that may counterbalance effector immune activation during checkpoint blockade. TGF-β-associated programs can contribute to stromal remodeling, immune exclusion, regulatory T-cell differentiation and impaired cytotoxic T-cell activity (43, 44). In peripheral blood, however, circulating TGF-β levels may not fully reflect tumor-site biology, and assay variability remains a substantial limitation. Its greatest potential may lie in composite models that integrate suppressive cytokine signals with effector-cell dynamics, myeloid markers and tumor-burden kinetics.

IL-17/type III activation, B-cell or plasmablast changes, autoantibody dynamics and eosinophilia form a toxicity-associated inflammatory and humoral module. IL-17A+ CD4+ T-cell expansion has been observed at irAE onset in melanoma (31); B-cell, plasmablast and autoantibody changes have been associated with irAEs in melanoma cohorts (34–36); and eosinophils or serum IFN-γ have been associated with irAEs in NSCLC (32, 38). These signals may indicate toxicity-prone inflammatory amplification but do not by themselves establish tumor-directed immunity.

Cytokine measurements are therefore most credible as time-resolved networks. Coordinated effector-cell activation with tumor-burden decline supports productive immunity; IL-8 elevation or failure to decline, and high IL-6 in selected gastric or esophageal cohorts, can accompany poorer outcomes (12, 33, 40, 42). By contrast, IL-17/type III activation with tissue-injury signals raises concern for toxicity in melanoma (31). Prospective studies should test predefined sampling windows and interpretable combinations rather than *post hoc* single-analyte thresholds.

## 6 The efficacy–toxicity interface: shared immune activation and divergent outcomes

Associations between irAEs and improved response or survival have been reported across several tumor types (7, 45, 46). These findings do not make irAEs surrogate markers of benefit: results are heterogeneous and vulnerable to immortal-time and treatment-exposure bias (47, 48). A more defensible interpretation is that efficacy, toxicity and resistance are clinically distinct outcomes that can arise from partially shared immune activation.

Checkpoint inhibition can reinvigorate T cells, promote effector trafficking, amplify cytokine networks and alter peripheral tolerance. Early circulating PD-1+ CD8+ responses are most informative when considered with tumor burden and clinical benefit (11, 25). When tolerance fails or inflammation is redirected, T-cell and IL-17-associated activation (24, 31), autoantibody changes (34, 36) and B-cell or plasmablast changes (35) can instead accompany tissue injury. Longitudinal T-cell changes associated with severe irAEs in melanoma support partial biological overlap, but do not establish that the same immune program caused tumor control (24).

Several features argue against treating irAEs as a proxy for efficacy. Toxicities differ by organ, timing, mechanism, reversibility and clinical consequence (6, 22, 23, 49). Severity also changes interpretation: mild events may coexist with continued therapy, whereas severe events can require treatment interruption and immunosuppression (46, 50). Circulating MDSCs and IL-8-related inflammation have been associated with poor outcomes or progression (26, 33), high IL-6 with poorer outcomes in gastric and esophageal cancers (40), and tumor-site TGF-β with immune exclusion and resistance (43). Analyses should therefore use organ- and severity-specific endpoints and account for the time patients must remain alive and exposed to treatment before an irAE can occur (47, 48).

## 7 Organ-specific immune biology of irAEs

Organ-specific irAEs are related but biologically distinct forms of checkpoint blockade-induced dysregulation (6, 23, 49, 51). Colitis can involve mucosal T-cell activation and TNF-related pathways (52), IL-17A+ CD4+ T cells in selected irAE tissue from melanoma patients (31), and microbiome-shaped responses (53). Pneumonitis in lung cancer can involve tissue-resident or infiltrating T cells, IFN-related trafficking and myeloid inflammation (54), while a high baseline eosinophil count has been associated with pneumonitis risk in NSCLC (38). Peripheral cytokines, eosinophils and circulating T-cell shifts can add context, but diagnosis still depends on severity, imaging, exclusion of infection and organ-specific assessment (5, 22, 55).

Cardiac, hepatic and endocrine toxicities further show why organ context matters. Myocarditis is uncommon but potentially fatal and can involve T-cell and macrophage infiltration, cytotoxic injury and proposed antigenic overlap between tumor and cardiac tissue (56). Hepatitis can reflect loss of hepatic tolerance and cytotoxic T-cell-mediated injury (57). Thyroiditis, hypophysitis, adrenalitis and diabetes-like presentations can involve tissue-restricted inflammation and, in some settings, pre-existing or emerging autoantibodies (6). Peripheral changes may inform systemic context, but organ-specific tests remain central to detection and management (5, 22, 55–58).

Peripheral-monitoring studies should therefore annotate irAEs by organ and severity. A single binary toxicity endpoint can obscure distinct clinical associations, as outcomes differ among skin toxicity, pneumonitis and severe irAEs in NSCLC (49).

Within this framework, peripheral immune profiles can be interpreted along two intersecting dimensions: productive anti-tumor activation and inflammatory or toxicity propensity. Immune-cold resistance may include delayed or absent early proliferating PD-1+ CD8+ responses (25), low invigoration relative to tumor burden (11), or unfavorable peripheral TCR features (13). Effective activation combines early CD8+ responses (11, 25), favorable or tumor-linked TCR changes (13, 29, 30) and declining circulating tumor DNA (ctDNA) or other tumor-burden markers (68–70). Toxicity-prone amplification includes toxicity-associated T-cell/TCR features (24, 39), IL-17/type III activation (31), B-cell or autoantibody changes (34–36), and eosinophil or IFN-γ associations in NSCLC (32, 38). Inflamed but ineffective resistance includes circulating MDSCs (26), IL-8-associated poor response or progression (33, 42), high IL-6 with poor outcomes in gastric or esophageal cancer (40), and tumor-site TGF-β immune exclusion (43, 44; ref. 44 is preclinical).

These patterns are overlapping interpretations rather than fixed biological states or validated patient categories. The same biomarker may therefore have different meanings depending on sampling time, accompanying immune signals, tumor-burden dynamics and evidence of organ injury. Prospective evaluation is required before the framework can inform treatment or surveillance decisions.

## 8 Cancer-type-specific considerations in peripheral immune monitoring

Peripheral immune profiles are unlikely to have the same meaning in every cancer. Evidence is most developed in melanoma and non-small cell lung cancer (NSCLC). In melanoma, circulating CD8+ reinvigoration and high-dimensional response profiles have been studied with anti-PD-1 therapy (11, 14), while toxicity-associated T-cell and IL-17/type III programs have been examined separately (24, 31). In NSCLC, eosinophils and serum IFN-γ have been associated with irAEs (32), baseline eosinophils with pneumonitis risk (38), and tissue or biomarker studies with pneumonitis biology and early detection (54, 59). Baseline lung disease, smoking, infection risk and complex radiographic findings remain important clinical confounders during pneumonitis assessment (5, 22, 55, 59).

Other tumors emphasize different monitoring layers. Peripheral TCR repertoire features have been associated with durable benefit in renal cell carcinoma, while circulating neoantigen-specific CD8+ responses may indicate tumor-directed activity in urothelial carcinoma (29, 30). In MSI-H/dMMR tumors, high antigenicity and neoantigen-driven T-cell responses support sensitivity to PD-1 blockade (10). In gastrointestinal settings, mucosal immunity, the microbiome and organ-specific toxicity may be particularly relevant (52, 53). These examples support stratification by tumor type, regimen, baseline burden, prior therapy and irAE phenotype rather than a universal blood-based model (60). **Supplementary Table 2** summarizes cancer-specific considerations.

## 9 Translating dynamic peripheral immune monitoring into clinical trials and practice

Translation requires candidate markers to progress from retrospective association to analytical validity, clinical validity and demonstrated clinical utility. Most circulating markers remain exploratory because sampling schedules, platforms, endpoints and statistical methods differ substantially across studies and syntheses (12, 26, 37). Development should begin with a defined clinical question, such as early response, emerging toxicity, resistance phenotype or rechallenge risk. **Table 2** lists the corresponding translational checkpoints.

**Table 2:** Translational checklist for dynamic peripheral immune monitoring.

Translational checkpoint	Why it matters	Proposed solution	Implementation endpoint	Representative references
Prespecified candidate sampling windows	Peripheral immune signals are time-dependent	Predefine baseline, early treatment, first imaging, irAE onset, progression and rechallenge windows	Comparable longitudinal datasets	Early response (11, 25); irAE monitoring (24, 31); rechallenge (61–63)
Biospecimen and assay documentation	Blood assays are sensitive to processing and platform variability	Report collection, processing, storage, assay methods and quality metrics transparently; use separate assay-specific standards when harmonization is required	Reproducible cross-center measurement	(64, 65)
Endpoint harmonization	Response, resistance and irAEs are inconsistently annotated	Use iRECIST or other prespecified response criteria, consensus resistance definitions and organ-/severity-specific irAE terminology	Interpretable clinical endpoints	Response (75); resistance (4); irAE terminology (23)
Bias-aware efficacy–toxicity analysis	irAE–outcome associations are vulnerable to immortal time bias	Apply landmark analyses, time-dependent models and sensitivity analyses	Valid inference at the efficacy–toxicity interface	(45, 47, 48)
Multi-marker model validation	High-dimensional immune data can overfit	Use prespecified features, external validation, calibration and transparent reporting	Robust integrated risk models	(66, 67, 71, 72)
Clinical utility testing	Association does not establish clinical usefulness	Use decision-curve analysis and prospective utility endpoints	Actionable biomarker thresholds	(73, 74)
Rechallenge risk stratification	Recurrent irAEs after treatment resumption remain difficult to predict	Integrate prior irAE phenotype, grade, resolution, immunosuppression and residual inflammation	Safer post-irAE treatment decisions	(61–63)
Multimodal implementation	Peripheral biomarkers alone do not capture tumor-local biology or imaging dynamics	Integrate tissue biomarkers, imaging, ctDNA and peripheral immune profiles	Biomarker-guided immunotherapy management	Tissue biomarker example (15); ctDNA monitoring (68, 70); integrated implementation remains prospective

irAE, immune-related adverse event; ctDNA, circulating tumor DNA.

Sampling schedules and assay requirements should be prespecified together. Pretreatment and early on-treatment blood assessments have been used for response monitoring (11, 25), while irAE onset and rechallenge require distinct clinical contexts (22, 61–63). Routine indices such as NLR, platelet-to-lymphocyte ratio and eosinophil counts have been studied as irAE-risk markers (27, 28), but they remain clinically nonspecific and sensitive to infection, corticosteroids, comorbidity and tumor burden. Specialized immune assays require assay-specific processing and quality control, and MDSC syntheses in particular call for marker and cutoff standardization (26). Specimen handling and assay methods should be reported transparently (64, 65). Feasibility, turnaround time, cost and cross-center reproducibility should be reported alongside statistical performance.

Clinical readiness depends on whether results can be delivered, interpreted and linked to a predefined action. Routine blood indices are accessible but nonspecific; specialized immune assays remain less standardized and may be limited by sample logistics, platform access and integration with radiology, laboratory and toxicity records. Action thresholds should specify when a result prompts intensified surveillance, confirmatory testing, resistance work-up, rechallenge counseling or trial stratification, and decision-curve analysis can evaluate whether a model adds net clinical benefit (73, 74). Biomarker and prediction-model studies should be reported transparently (64, 66). Reimbursement and regulatory expectations also require consideration.

Multi-marker models may be preferable because checkpoint blockade affects several immune layers at once. Complementary inputs include inflammatory indices, effector T-cell and suppressive myeloid dynamics, soluble signals, TCR features and circulating tumor DNA as an orthogonal measure of tumor burden (11, 12, 26, 30, 68–70). Models require prespecified features, external validation, calibration and transparent reporting, together with safeguards against overfitting and evidence that predictions improve decisions beyond standard assessment (66, 67, 71–74).

Clinical utility requires prospective biomarker-guided trials with predefined sampling, harmonized immunotherapy response criteria (75), consensus resistance definitions (4), organ- or severity-specific irAE terminology (23), and bias-aware analyses (47, 48). Rechallenge studies should account for the prior irAE, its grade and resolution, immunosuppression and residual organ vulnerability (61–63), while landmark or time-dependent methods are needed when relating toxicity to efficacy (47, 48). Until these requirements are met, peripheral monitoring should remain an adjunct to clinical assessment, tissue biomarkers and imaging. **Supplementary Figure 1** outlines the stepwise development path.

## 10 Conclusions and future directions

Peripheral immune biomarkers add a longitudinal view of host immunity to tumor-centered assessment. Their value lies less in any isolated measurement than in relating changes in immune cells, soluble mediators and tumor burden to treatment timing and clinical events.

This review uses four heuristic patterns to organize those trajectories: immune-cold resistance, effective anti-tumor activation, toxicity-prone inflammatory amplification and inflamed but ineffective resistance. The patterns describe overlapping interpretations of systemic immune remodeling, not validated biological states or patient categories. The next step is prospective testing of clearly defined clinical questions. Studies should prespecify sampling windows, assays, organ- and severity-specific irAE endpoints, tumor-burden measures and bias-aware analyses. Candidate models must also demonstrate calibration, external validity and useful action thresholds. Until then, peripheral readouts should remain complementary to clinical, radiographic and tissue-based assessment.

## Supplementary material

Supplementary Figure 1 Roadmap for translating dynamic peripheral immune monitoring into biomarker-guided immunotherapy.
