# Supplementary material for: Dynamic peripheral immune monitoring during checkpoint blockade: a framework for interpreting response, toxicity and resistance
Source: Front Immunol. 2026 Aug 3;17:1902709. doi: 10.3389/fimmu.2026.1902709 (PMC13479434; doi:10.3389/fimmu.2026.1902709)
Supplement: Supplementary file 1 [file Table1.docx]

**Supplementary Table 1.** Tissue biomarkers and limitations for systemic immune-state monitoring

Tissue biomarkers and limitations for systemic immune-state monitoring. This supplementary table summarizes the value and limitations of established and emerging tissue-based biomarkers used in immunotherapy and explains why dynamic peripheral immune monitoring should be viewed as complementary to tissue-centered biomarkers.

| **Tissue biomarker / domain** | **Main value** | **Key limitation** | **Complementary role of peripheral monitoring** | **Representative references** |
| --- | --- | --- | --- | --- |
| PD-L1 expression | Captures local adaptive immune resistance and supports patient selection in selected tumor types and regimens | Predictive performance varies across cancer types and treatment settings; PD-L1 and TMB provide partly independent information | Tracks host immune competence and systemic immune remodeling beyond the sampled lesion | (8, 16) |
| Tumor mutational burden | Captures tumor antigenic potential and can enrich for benefit in selected TMB-high settings | Antigenic potential does not ensure productive immunity | Indicates whether antigenic potential translates into systemic immune activation | (9, 16-18) |
| MSI-H/dMMR status | Captures hypermutation and immune visibility from mismatch-repair deficiency, with strong predictive value in selected solid tumors | Applies to a limited subset and does not capture toxicity propensity | Helps contextualize response kinetics and systemic immune activation | (10) |
| TILs and tertiary lymphoid structures | Capture local immune infiltration and organized immune niches associated with ICI sensitivity | Spatial heterogeneity; limited temporal resolution; non-standardized assessment | Tracks treatment-induced effector, humoral and cytokine dynamics over time | (19-21) |
| IFN-γ/T-cell-inflamed signatures | Capture pre-existing cytotoxic activity and chemokine signaling linked to PD-1/PD-L1 sensitivity | Tumor-site inflammation may not reflect systemic immune state | Helps distinguish productive effector activation from inflammatory resistance or toxicity | (8, 15) |
| TGF-β-associated tissue resistance | Captures suppressive stromal programs and T-cell exclusion that may explain resistance | Repeat biopsies are invasive and lesion-specific | Helps distinguish immune-cold escape from inflamed but ineffective resistance when paired with blood and ctDNA | (43, 44) |
| Tissue biomarkers as a group | Capture tumor immunogenicity and local immune contexture for tumor-centered treatment selection | Static, spatially constrained and incomplete for systemic remodeling | Complements dynamic peripheral readouts of immune-state transitions | (8-10, 15) |
