# Supplementary material for: Dynamic peripheral immune monitoring during checkpoint blockade: a framework for interpreting response, toxicity and resistance
Source: Front Immunol. 2026 Aug 3;17:1902709. doi: 10.3389/fimmu.2026.1902709 (PMC13479434; doi:10.3389/fimmu.2026.1902709)
Supplement: Supplementary file 2 [file Table2.docx]

**Supplementary Table 2. Cancer-type-specific considerations for dynamic peripheral immune monitoring during checkpoint blockade.**

This supplementary table summarizes tumor-type-specific similarities and differences that may influence the interpretation of peripheral immune biomarkers during checkpoint blockade. These considerations are intended to guide cancer-type-aware validation rather than define tumor-specific clinical decision rules.

| **Cancer type or setting** | **Relevant peripheral monitoring signals** | **Distinct clinical considerations** | **Interpretation caveats** | **Representative references** |
| --- | --- | --- | --- | --- |
| Melanoma | CD8+ T-cell reinvigoration; high-dimensional immune profiles; IL-17/type III toxicity signals | Strong evidence base for response- and irAE-associated peripheral immune findings | Findings may not generalize to less immunogenic tumors or different ICI regimens | (11, 14, 24, 31) |
| Non-small cell lung cancer | NLR, eosinophils, cytokines and pneumonitis-related immune signals | Lung comorbidity, smoking history, infection and radiographic overlap are important | Inflammation may reflect tumor burden, infection or lung injury rather than tumor-directed immunity | (32, 38, 54, 59) |
| Renal cell carcinoma | TCR repertoire features and clonal dynamics | Durable benefit may be linked to peripheral repertoire features | Tumor specificity requires validation and ideally tumor-blood clonal linkage | (29) |
| Urothelial carcinoma | Neoantigen-specific circulating CD8+ T-cell responses | Peripheral tumor-reactive T cells may support response interpretation | Requires antigen-specific validation or tumor-blood linkage | (30) |
| Gastrointestinal settings | Mucosal immune signals, microbiome-related features and inflammatory markers | Colitis, hepatitis and microbiome effects may influence monitoring | Peripheral markers should be interpreted with organ-specific findings and tumor-burden dynamics | (52, 53) |
| MSI-H/dMMR tumors | High antigenicity and neoantigen-driven T-cell responses | Strong tumor immunogenicity; MSI-H/dMMR status does not itself define toxicity risk | Tissue context, tumor burden dynamics and irAE phenotype remain essential | (10, 60) |
